# Transcatheter Versus Minimally Invasive Surgical Aortic Valve Replacement With Rapid-Deployment Valves: A Propensity-Matched Analysis

**DOI:** 10.1093/icvts/ivag007

**Published:** 2026-02-17

**Authors:** Paul Werner, Martin Winter, Christoph Krall, Raphael Rosenhek, Amila Kahrovic, Alfred Kocher, Daniel Zimpfer, Martin Andreas, Iuliana Coti

**Affiliations:** Department of Cardiac and Thoracic Aortic Surgery, Medical University of Vienna, 1090 Vienna, Austria; Department of Cardiac and Thoracic Aortic Surgery, Medical University of Vienna, 1090 Vienna, Austria; Center for Medical Data Science, Medical University of Vienna, 1090 Vienna, Austria; Department of Cardiology, Medical University of Vienna, 1090 Vienna, Austria; Department of Cardiac and Thoracic Aortic Surgery, Medical University of Vienna, 1090 Vienna, Austria; Department of Cardiac and Thoracic Aortic Surgery, Medical University of Vienna, 1090 Vienna, Austria; Department of Cardiac and Thoracic Aortic Surgery, Medical University of Vienna, 1090 Vienna, Austria; Department of Cardiac and Thoracic Aortic Surgery, Medical University of Vienna, 1090 Vienna, Austria; Department of Cardiac and Thoracic Aortic Surgery, Medical University of Vienna, 1090 Vienna, Austria

**Keywords:** aortic valve replacement, minimally invasive, rapid-deployment, transfemoral transcatheter aortic valve replacement, propensity score matching

## Abstract

**Objectives:**

This study evaluates intermediate-term survival and valve-related complications in patients undergoing minimally invasive surgical aortic valve replacement (MI-SAVR) using rapid-deployment (RD) valves compared with those receiving transfemoral transcatheter aortic valve replacement (TF-TAVR) after propensity-matched analysis.

**Methods:**

All consecutive patients treated with either isolated MI-SAVR with an RD valve or TF-TAVR at a single cardiac-surgery centre were retrospectively reviewed. A propensity score was created, and exact matching was applied after the maximum propensity score difference. Nearest-neighbour matching was conducted with a caliper of 0.2 standard deviations of the logit of the propensity score, without replacement and with a 1:1 matching ratio.

**Results:**

From April 2011 to June 2022, 926 patients underwent either isolated MI-SAVR with an RD valve (*n* = 400) or TF-TAVR (*n* = 526). After propensity score matching, the final cohort (*n* = 366) included 183 matched pairs. Operative mortality was 0% after MI-SAVR compared with 3.3% (*n* = 6) following TF-TAVR (*P* = .03). Perioperative stroke occurred in 2.7% (*n* = 5, MI-SAVR) vs 2.2% (*n* = 4, TF-TAVR, *P* = 1). At 3 years, MI-SAVR was associated with significantly lower rates of paravalvular leakage (2.2% vs 13.8%, *P* < .001), new pacemaker implantations (6.6% vs 14.8%, *P* = .01) and a composite end-point of thromboembolic and major bleeding events (7.2% vs 12.7%, *P* = .025). No difference between aortic valve re-interventions and stroke was identified between groups. Survival at 1- and 3-year follow-up was 98% and 88% (MI-SAVR) and 88% and 67% (TF-TAVR) respectively (*P* < .001). EuroScore II emerged as an independent predictor of mortality (HR 1.12 [1.02, 1.23], *P* = .014).

**Conclusions:**

Minimally invasive SAVR with RD-valves could represent a treatment modality to TF-TAVR for severe AS in an older, low-risk patient cohort. In our retrospective cohort study, MI-SAVR was linked to improved survival and lower rates of permanent pacemaker implantation and paravalvular leakage.

## Graphical abstract

**Figure:**
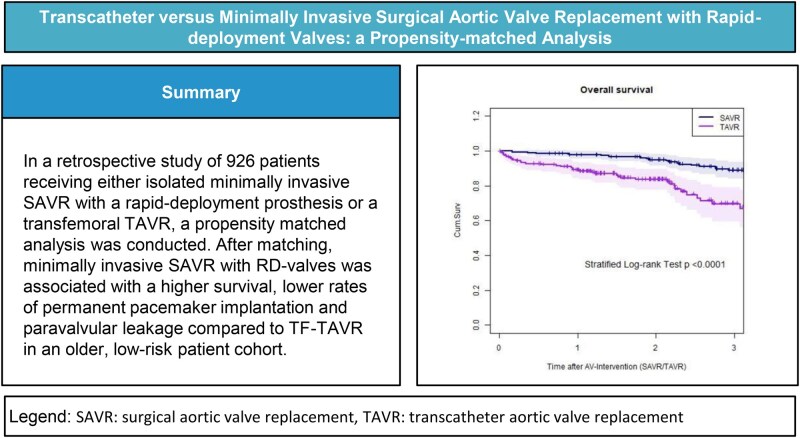

## INTRODUCTION

European guidelines recommend transfemoral transcatheter aortic valve replacement (TF-TAVR) for patients with severe aortic stenosis (AS) above the age of 70 years, irrespective of surgical risk.^1^ These patients were just recently considered a prime target for minimally invasive aortic valve replacement (MI-SAVR) with rapid-deployment (RD) prostheses.^2^

Rapid-deployment valves such as the Intuity (Edwards Lifesciences LLC, Irvine, CA, United States) were developed on the basis of conventional sutured bioprostheses and the stent technology of modern transcatheter valves. They require fewer implantation sutures, which facilitates the implantation and shortens procedural times. Clinical data showed improved haemodynamics of RD valves in direct comparison to other bioprostheses, possibly attributable to the omission of pledgeted sutures in the left ventricular outflow tract.^3^^,^^4^ These made RD valves an ideal match for minimally invasive valve replacement, which is technically more demanding and associated with longer procedural times, yet might benefit the patients due to a reduction in surgical trauma.

Results from major randomized trials comparing TAVR to SAVR demonstrated non-inferiority or even superiority of TAVR at 1-year follow-up, leading to a paradigm shift in AS treatment.^5^^,^^6^ Besides the limited follow-up, concerns were raised if some of those trials were depicting a real-world population, as exclusion criteria were numerous and strict. After the approval of TAVR in low-risk patients, the number of younger TAVR patients has risen drastically. In the U.S., TAVR increased more than 2.7-fold in patients <65 years since 2015 and is now nearly equal in volumes as SAVR.^7^ Although randomized trials have proven TAVR to be non-inferior in the early follow-up, recent studies question its superiority in low-risk patients when it comes to longer follow-up.^8^^,^^9^ Most randomized controlled trials mainly utilized conventional sutured bioprostheses for SAVR implanted over a median sternotomy; therefore, the current literature lacks data comparing minimally invasively implanted RD valves versus TAVR. Given the reduced invasiveness of MI-SAVR, a direct comparison with TAVR is of great interest for the surgical and interventional community.

## METHODS

### Patients

Between April 2011 and June 2022, all consecutive patients undergoing isolated minimally invasive surgical aortic valve replacement (MI-SAVR) with a rapid-deployment valve (Intuity Elite System, Edwards Lifesciences LLC, Irvine, CA, United States) or transfemoral transcatheter aortic valve replacement (TF-TAVR) using balloon or self-expanding valves at a single institution were analyzed. Patients treated with a conventional sutured valve, full sternotomy, concomitant procedures, or non-transfemoral TAVR were excluded. A 1:1 propensity score matching (PSM) was created with 18 parameters (**Table 1**) and the final cohort included 183 pairs (**Figure S1**). Femoral access was guided by digital subtraction angiography or echocardiography, with a later shift to echo-guided puncture. Main access was closed using 2 endovascular devices (Perclose ProGlide/ProStyle, Abott Laboratories); secondary access with a 6 F Angio-Seal. Cerebral embolic protection (CEP) was introduced in late 2019 and became the standard of treatment in anatomically suitable patients.

**Table 1.** Baseline Patient Characteristics

Matching parameters	Unmatched cohort (*n* = 926)			PS-matched cohort (*n* = 366)		
	MI-SAVR (*n* = 400)	TF-TAVR (*n* = 526)	*P*-value	MI-SAVR (*n* = 183)	TF-TAVR (*n* = 183)	*P*-value
Age (years, SD)	72.6 (7.4)	80.1 (7.2)	<.001	76 (7.2)	77 (7.5)	.23
Sex (male, %)	201 (50.3)	267 (50.9)	.89	92 (50.3)	88 (48.1)	.754
EuroSCORE II (IQR)	1.4 (1.0-2.2)	3.9 (2.3-6.1)	<.001	1.8 (1.3-3.4)	2.2 (1.7-3.5)	.002
BMI (kg/m^2^, SD)	28.2 (5.3)	27.3 (5.4)	.007	27.6 (5.0)	27.8 (5.4)	.76
Creatinine (mg/dL, SD)	1.0 (0.4)	1.3 (0.9)	<.001	1.0 (0.39)	1.1 (0.84)	.51
Diabetes (%)	99 (24.8)	158 (30.1)	.076	49 (26.8)	49 (26.8)	1
Periph. vascular disease (%)	18 (4.5)	56 (10.6)	<.001	9 (4.9)	11 (6)	.82
Prior MI (%)	17 (4.3)	41 (7.8)	.029	9 (4.9)	10 (5.5)	1
Atrial fibrillation (%)	53 (13.3)	189 (36)	<.001	40 (21.9)	43 (23.5)	.8
Porcelain aorta (%)	0	45 (9.2)	<.001	0	0	1
Arterial hypertension (%)	339 (84.8)	474 (90.1)	.011	158 (86.3)	162 (88.5)	.64
Dyslipidaemia (%)	235 (58.8)	426 (81.0)	<.001	131 (71.6)	132 (72.1)	1
Smoking history (%)	70 (17.5)	135 (25.7)	.003	36 (19.7)	37 (20.2)	1
Prior stroke (%)	62 (15.5)	56 (10.6)	.036	23 (12.6)	23 (12.6)	1
Prior PCI (%)	33 (8.3)	139 (26.4)	<.001	22 (12.0)	24 (13.1)	.875
Previous pacemaker (%)	18 (4.5)	71 (13.5)	<.001	15 (8.2)	14 (7.7)	1
COPD (%)	63 (15.8)	97 (18.4)	.293	29 (15.8)	30 (16.4)	1
Renal replacement therapy (%)	3 (0.8)	15 (2.8)	.028	2 (1.1)	3 (1.6)	1

Abbreviations: BMI, body mass index; COPD, chronic obstructive pulmonary disease; EuroSCORE II, European System for Cardiac Operative Risk Evaluation; MI, myocardial infarction; MI-SAVR, minimally invasive surgical aortic valve replacement; PS, propensity score; TF-TAVR, transfemoral transcatheter aortic valve implantation.

Data were collected retro- and prospectively as previously reported.^2^ All consecutive patients who underwent TAVR starting from September 2017 in the surgical lead TAVR programme were enrolled in the ongoing prospective “Victory II Registry”. Both registries were approved by the Ethical Committee of the Medical University of Vienna (1861/2016 and 1680/2020) and all patients signed written informed consent.

### Study endpoints

The primary endpoint was overall survival; operative mortality included 30-day and in-hospital mortality. Secondary endpoints were perioperative stroke and embolization events, bleeding events, non-structural valve dysfunction (NSVD), aortic valve re-interventions (including all re-operations or valve-in-valve procedures due to structural valve degeneration [SVD], NSVD, valve thrombosis, and valve endocarditis), and postoperative permanent pacemaker implantations (PPI) ≤14 days. Perioperative stroke was defined as a new onset of a neurological deficit lasting longer than 24 h within the first 72 h after the procedure. All embolic neurological events after these 72 h lasting longer than 24 h were defined as embolization events (except intracranial bleedings). Bleeding events were defined as bleeding requiring medical intervention, hospitalization, or red blood cell (RBC) transfusions postoperatively. Perioperative RBC transfusions were stated separately. All adverse events were classified after the EACTS/STS/AATS guidelines for reporting morbidity and mortality after valve interventions in accordance with the VARC 3 criteria.^10^^,^^11^ The valve-related adverse events were reported at 1- and 3-year follow-up, and the cumulative incidence (CI) rates were estimated, accounting for death as a competing event as reported.

### Statistical analysis

Propensity scores were calculated using a logistic regression model with the covariates defined in **Table 1**. All baseline parameters included in the propensity score matching were complete. Nearest-neighbour matching was conducted with a caliper of 0.2 standard deviations of the logit of the propensity score, without replacement, and with a 1:1 matching ratio. Standardized mean differences before and after matching and overlap are reported (**Figures S2 and S3**). Wilcoxon signed-rank tests respectively McNemar tests were performed to compare matched groups with respect to covariables.

Continuous data are presented as mean and standard deviations (SD) or median (IQR: 25th-75th interval); categorical variables as counts and percentages. Survival was estimated using Kaplan-Meier analysis with log-rank testing. Competing risk analyses with a Fine–Gray model using cluster-robust variance were performed. Competing risk models were analyzed for aortic valve reintervention, paravalvular leakage (PVL), stroke, major bleeding, pacemaker implantation (≤14 days), and the composite thromboembolic or bleeding event. For competing risk analyses, risk differences and ratios for 1 and 3 years have been added. Confidence intervals for RD/RR and RMST were obtained via matched-pair (MatchID) cluster bootstrap (*B* = 1000). A 2-sided *P* value of <.05 was considered to be statistically significant. Analysis was done using R, version 4.2.2.

## RESULTS

A total of 926 consecutive patients who underwent either isolated minimally invasive surgical aortic valve replacement (MI-SAVR) with a rapid-deployment valve (Intuity Elite System, Edwards Lifesciences LLC, Irvine, CA, United States) (*n* = 400) or transfemoral transcatheter aortic valve replacement (TF-TAVR) with different balloon or self-expanding transcatheter valves (*n* = 526) were selected for this analysis. Following exact matching after maximum propensity score difference, the final cohort (*n* = 366) included 183 matched pairs. Unmatched TAVR patients were significantly older: MI-SAVR 72.6 (7.4) years vs TF-TAVR 80.1 (7.2) years, *P* < .001 and presented with a higher rate of comorbidities (**Table 1**). Baseline characteristics were well balanced after matching; however, patients treated with TF-TAVR had a slightly but significantly higher EuroScore II (1.8% [IQR: 1.3% to 3.4%] vs 2.2 [IQR: 1.7% to 3.5%]; *P* = .002). The distribution of low (EuroScore II 0 to 4%), medium (4%-8%), and high (>8%) surgical risk patients did not differ between groups (*P* = .08).

Patients undergoing minimally invasive RD-SAVR were operated either through an upper-hemisternotomy (*n* = 82, 44.8%) or through a right anterior thoracotomy (*n* = 101, 55.2%) and underwent mostly central arterial and venous canulation (*n* = 179, 97.8%); there were only 2 conversions to full sternotomy in cases of thoracotomies due to bleeding. Besides the longer operative times, patients in the SAVR group had a mean perfusion and clamp times of 106 (25) min and 70 (20) min, respectively.

Several different third-generation transcatheter valves were implanted (Abbott Portico/Navitor 38.1%, Medtronic Evolut Pro/Pro Plus 30.9%, Edwards Sapien S3/Ultra 27.6%, Symetis Accurate Neo 2.2%, Allegra THV 1.1%). A total of 124/183 (68%) patients underwent TAVR with a CEP device (either Sentinel: *n* = 114 or TriGUARD: *n* = 10); after the introduction of CEP in our clinical practice (October 2019), 87% of our TAVR cohort underwent intervention with a CEP device. A total of 42/132 (32%) patients implanted with a self-expanding TAVR required post-dilation.

Operative mortality was 0% (MI-SAVR) and 3.3% (*n* = 6, TF-TAVR, *P* = .04), respectively (**Table 2**). Perioperative stroke was observed in 2.7% (*n* = 5, MI-SAVR) vs 2.2% (*n* = 4, TF-TAVR, *P* = 1). New early (≤14-days) PPI was more common after TF-TAVR (13.7%, *n* = 25) than after MI-RD-SAVR (7.1%, *n* = 13, *P* = .052). More patients required at least one RBC transfusion in the MI-SAVR group (23.0% vs 11.5%, *P* = .006), although the number of patients requiring ≥2 RBC units did not differ between groups (3.3% vs 5.5%, *P* = .45) and the mean number of blood transfusions per patient did also not differ (SAVR 0.39 vs TAVR 0.33, *P* = .566). Acute kidney injury did not differ between groups (4.9% vs 9.8%, *P* = .11) and major vascular complications occurred only in patients undergoing TF-TAVR (3.3% vs 0%, *P* = .04). Non-structural valve dysfunction (NSVD) was also higher in this patient cohort, with 1.6% of patients in MI-SAVR (*n* = 3) vs 8.2% of patients in TF-TAVR (*n* = 15, *P* = .01). A total of 29/183 (16%) patients had a degree of primary access closure device failure with bleeding: out of these, in 23 (79%) patients the primary access was additionally closed with an 8-french Angio-Seal, 4 (14%) patients underwent crossover balloon dilatation, and surgical conversion was necessary only in 2 cases (7%).

**Table 2.** In-Hospital and 30-Day Early Outcomes After PS Matching for Patients Treated With RD MI-SAVR Versus TF-TAVR

	MI-SAVR	TF-TAVR	*P*-value
	(*n* = 183)	(*n* = 183)	
Operative mortality (%)	0 (0)	6 (3.3)	.041
Periprocedural stroke (%)	5 (2.7)	4 (2.2)	1
Periprocedural TIA (%)	2 (1.1)	2 (1.1)	1
Blood transfusions ≥2 RBC (%)	6 (3.3)	10 (5.5)	.453
Vascular complications (%)	0 (0)	6 (3.3)	.041
Pacemaker implantation < 14 days (%)	13 (7.1)	25 (13.7)	.052
Acute kidney injury (%)	9 (4.9)	18 (9.8)	.11
Perioperative myocardial infarction (%)	0	0	1
Moderate-severe PVL at discharge (%)	3 (1.6)	15 (8.2)	.01
Procedural time (min, SD)	220 (44)	63 (37)	<.001
Hospital stay (days), IQR	8 [7-12]	3 [2-5]	<.001

Wilcoxon signed-rank tests resp. McNemar tests were performed.

Abbreviations: IQR, interquartile range; RBC, red blood cells; SD, standard deviation; TIA, transient ischaemic attack.

### Survival

**Figure 1.**
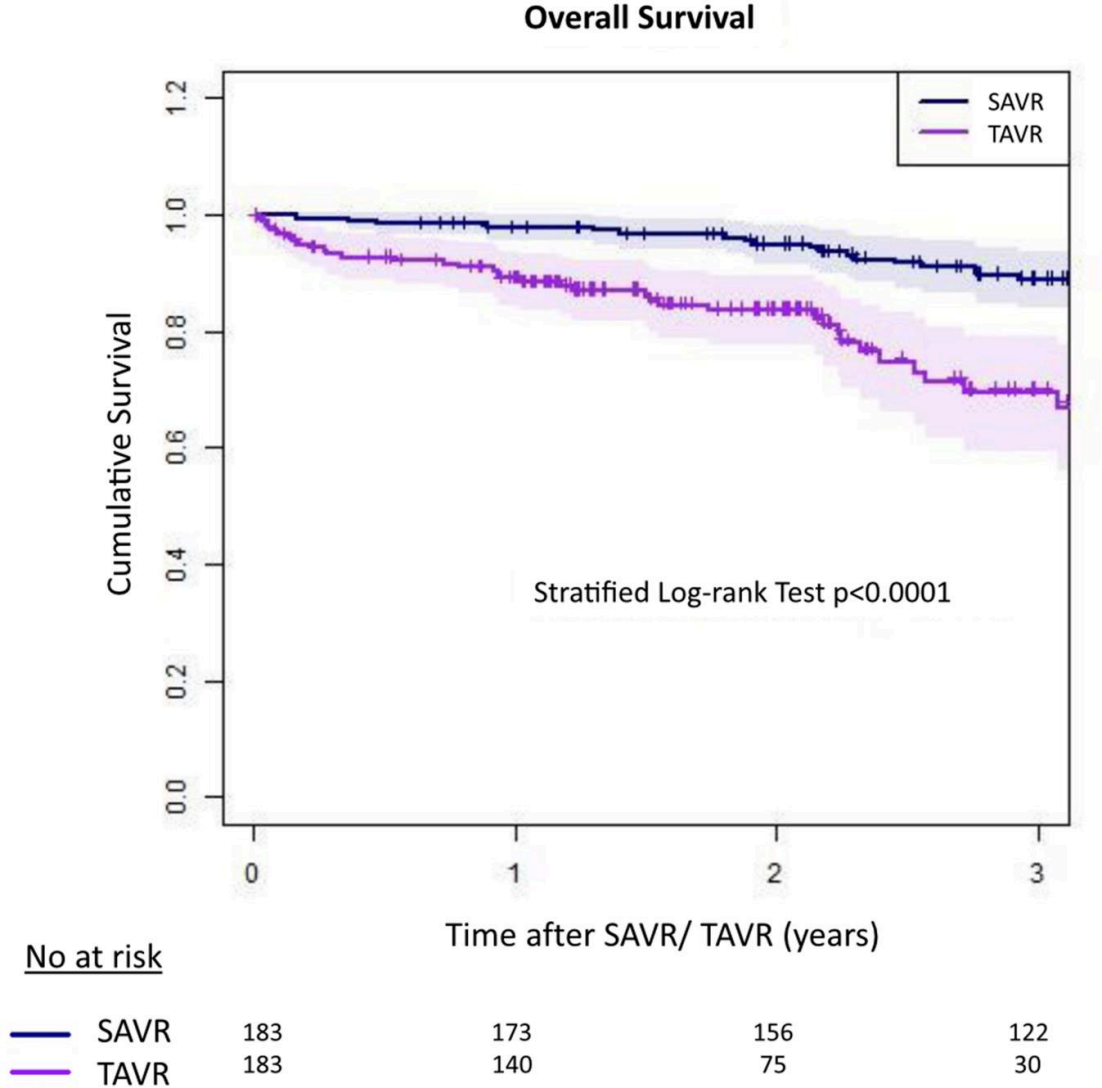
Kaplan–Meier Survival Curve. Survival in patients with MI-SAVR is significantly higher than in patients with TAVR—log-rank *P* < .001.

Survival at 1 and 3-year follow-up was 98% ± 1% and 88% ± 3% for MI-SAVR and 88% ± 1% and 66% ± 5% for TF-TAVR (log-rank test *P* < .001), respectively (**Figure 1**).

The absolute difference in overall survival was −9.3 percentage points at 1 year (RD = −0.093; 95% CI: −0.130 to −0.054) and −20.1 percentage points at 3 years (RD = −0.201; 95% CI: −0.297 to −0.113). Corresponding survival ratios were RR for 1 year = 0.905 (95% CI: 0.866-0.945) and RR for 3 years = 0.772 (95% CI: 0.672-0.870). The difference in restricted mean survival time over 3 years (tau = 1095 days) was −130 days.

### Valve dysfunction, PPI, and thromboembolic and bleeding events

**Figure 2.**
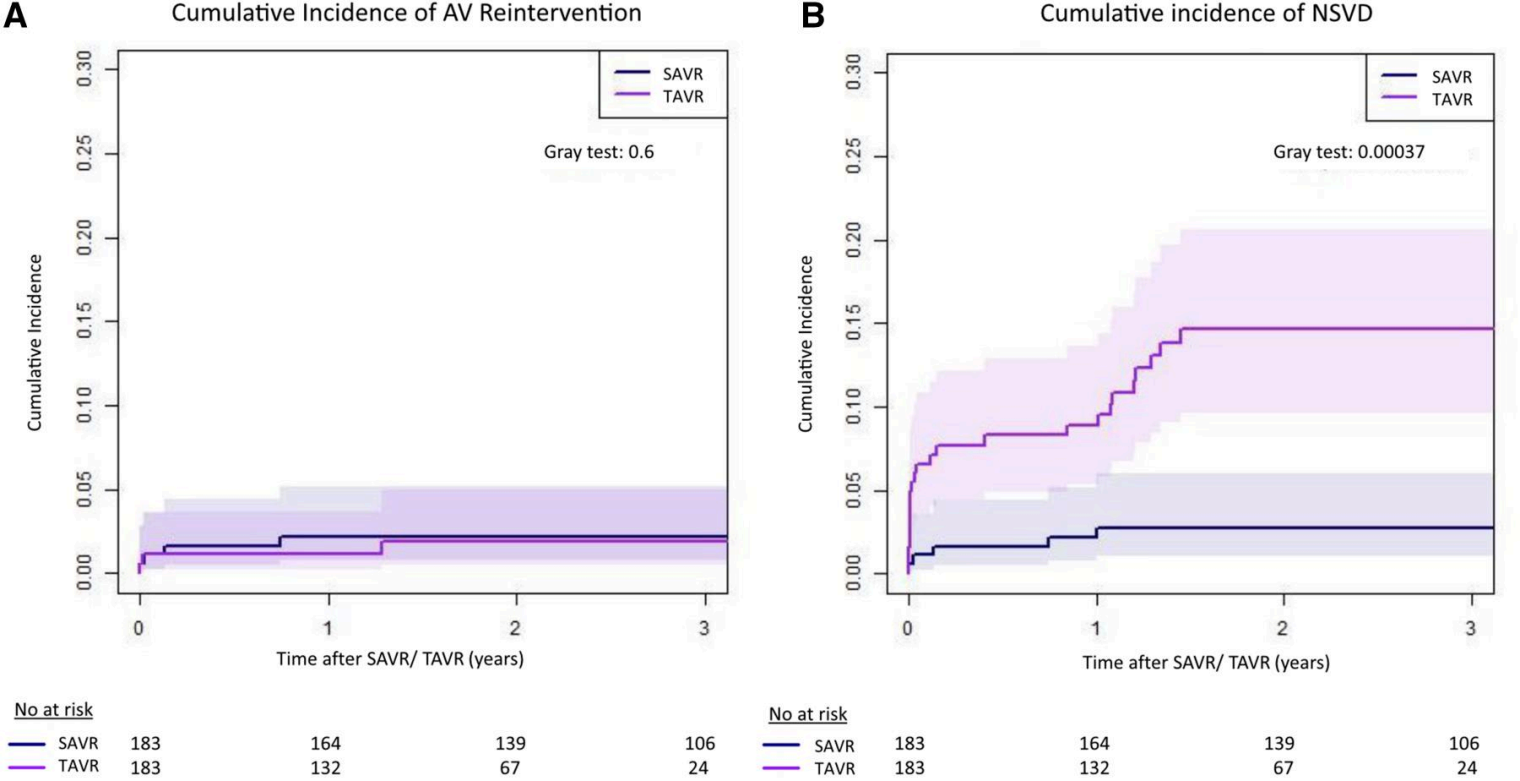
Cumulative Incidence Curves for Prosthetic Failure. (A) Re-operation with valve explanation or reintervention. (B) Non-structural valve dysfunction (NSVD). Competing risk analyses were performed to estimate the cumulative incidence considering death as a competing event.

A composite endpoint of reoperation with valve explantation or valve-in-valve procedure occurred in 1.6% (95% CI: 0.5%-4.4%) after MI-RD-SAVR and 1.1% (95% CI: 0.2%-3.6%) after TF-TAVR at 3-year follow-up (*P* = .712, **Figure 2A**, **Table 3**). In total, 3 patients in the TAVR cohort underwent valve re-interventions with surgical valve replacement due to severe paravalvular leak (*n* = 2) on day 7 and 16 months after the index procedure, and the third due to ventricular septal defect on POD 1. In the SAVR group, 6 patients underwent re-interventions on the study valve during the follow-up: 2 due to structural valve degeneration (one valve-in-valve at 51 months after SAVR and one surgical reoperation at 103 months after the index procedure); another 4 patients in the RD-SAVR group underwent reoperation with valve explantation due to severe paravalvular leak at day 1, day 7, day 48, month 9 after the index procedure. No cases of valve thrombosis or endocarditis requiring valve explantation were registered.

**Table 3.** Valve Related Adverse Events at 1- and 3-Year Follow-Up

Variables	MI-SAVR (*N* = 183)	TAVR (*N* = 183)	Risk differences	Risk ratio	*P* value (Gray test)
	(95% CI)	(95% CI)			
Composite AV Re-op					
1 year	1.6% (0.5%-4.4%)	0.6% (0.1%-2.8%)	−0.05 (0.1, 0.01)	0.28 (0,1.66)	.6
3 years	1.6% (0.5%-4.4%)	1.1% (0.2%-3.6%)			
NSVD					
1 year	1.6% (0.5%-4.4%)	8.3% (4.8%-12.9%)	0.1 (0.05, 0.14)	3.14 (1.84,7.93)	<.001
3 years	2.2% (0.7%-5.2%)	13.8% (9.0%-19.7%)			
Pacemaker implantation					
1 year	6.6% (3.6%-10.8%)	14.8% (10.1%-20.3%)	0.07 (0.01, 0.12)	1.77 (1.11, 3.11)	.01
3 years	6.6% (3.6%-10.8%)	14.8% (10.1%-20.3%)			
Thromboembolic and bleeding events					
1 year	6.0% (3.2%-10.1%)	9.0% (5.4%-13.8%)	0.63 (0.03, 0.71)	8.91 (1.31, 14.16)	.053
3 years	7.2% (4.0%-11.7%)	12.7% (7.9%-18.7%)			
Stroke					
1 year	2.7% (1.0%-5.9%)	2.2% (0.7%-5.2%)	0.02 (−0.03, 0.07)	1.44 (0.44, 3.43)	.84
3 years	3.3% (1.4%-6.6%)	2.8% (1.1%-6.0%)			
Major bleeding					
1 year	0.6% (0.1%-2.8%)	4.0% (1.8%-7.6%)	0.64 (0.02, 0.72)	27.37 (1.6, 73.92)	.023
3 years	1.7% (0.5%-4.6%)	6.1% (2.8%-11.4%)			

The cumulative incidence (CI) rates are estimated, accounting for death as a competing event. The cumulative incidence rates at 3 years are estimated, accounting for death as a competing event. The Gray’s test was used to test for differences in the cumulative incidence curves.

Abbreviations: AV Re-op, composite end-point of aortic valve reoperation with valve explantation or valve-in-valve procedures; NSVD, non-structural valve dysfunction.

Moderate-severe NSVD was higher after TAVR with 13.8% (95% CI: 9.0%-19.7%) compared with RD-SAVR with 2.2% (95% CI: 0.7%-5.2%) at 3-year follow-up (*P* < .001, **Figure 2B**). Moreover, the subgroup of patients implanted with a self-expanding TAVR was more prone to moderate-severe postoperative paravalvular leak (NSVD), compared with balloon-expandable valves (15% vs 8%, *P* = .05).

**Figure 3.**
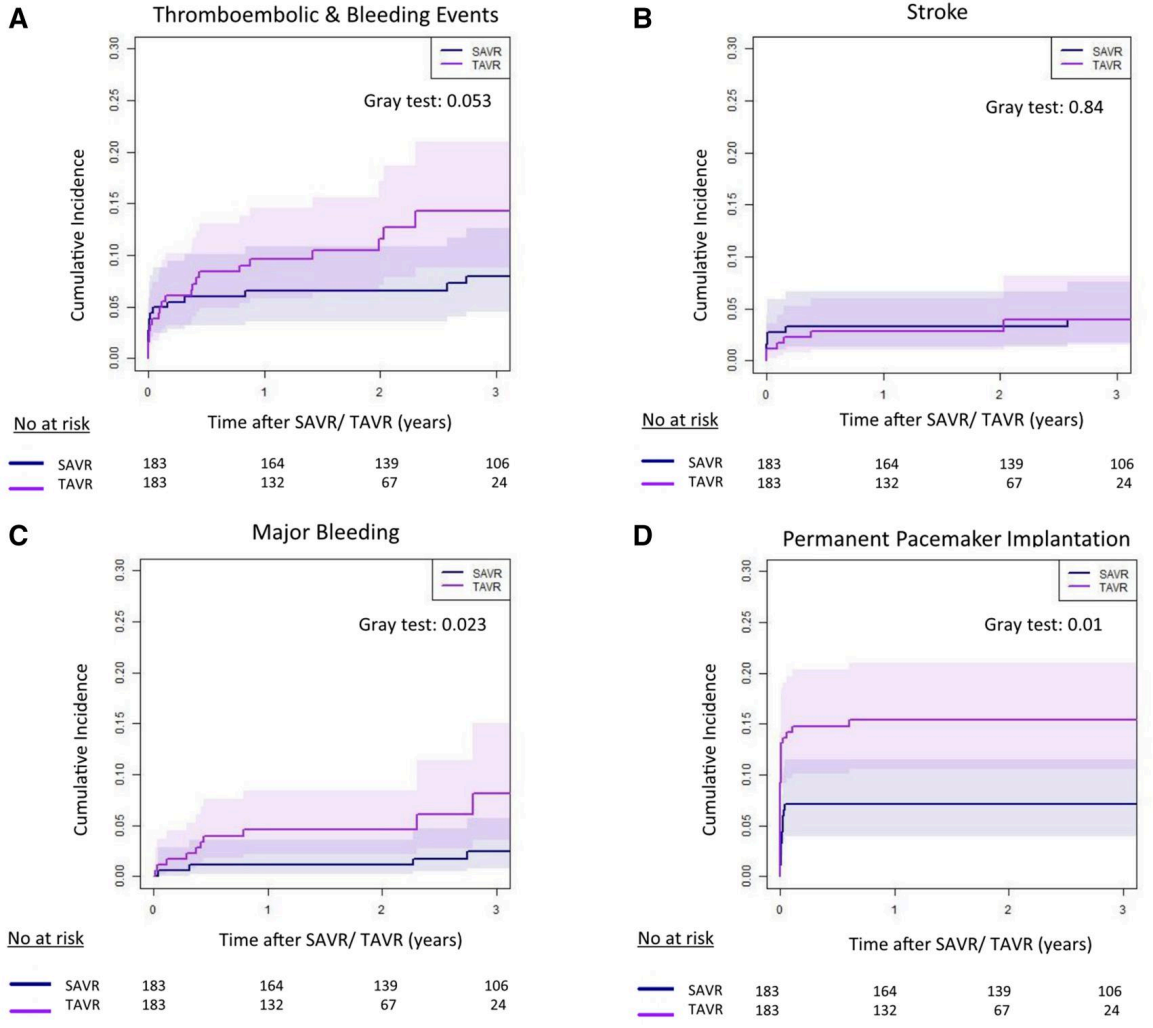
Cumulative Incidence of the Composite Endpoint Thromboembolic and Major Bleeding Events (A), Stroke (B), Major Bleeding (C), and Permanent Pacemaker Implantation (D). Competing risk analyses were performed to estimate the cumulative incidence, considering death as a competing event.

The cumulative incidence of thromboembolic and bleeding events at 3-year follow-up was higher after TAVR with 12.7% (95% CI: 7.9%-18.7%) compared with MI-RD-SAVR with 7.2% (95% CI: 4.0%-11.7%, *P* = .025, **Figure 3**), with a higher rate of major bleeding events (*P* = .006, **Figure 3**). No difference was identified for the cumulative incidence of stroke (*P* = .778, **Figure 3**). The rate of PPI at 3-year follow-up was higher after TF-TAVR with 14.8% (95% CI: 10.1%-20.3%), compared with RD MI-SAVR with 6.6% (95% CI: 3.6%-10.8%, *P* = .01, **Figure 3**).

## DISCUSSION

This study examined the early and intermediate-term outcomes of patients with AS who underwent TF-TAVR or MI-SAVR in 2 PSM cohorts. The main findings are: (1) Operative and mid-term survival was higher in the RD MI-SAVR group, a pattern observed in other non-randomized studies (2) Vascular complications, NSVD, PPI, and a composite end-point of major bleeding and thromboembolic events were more frequent after TF-TAVR.

Treatment of AS has evolved markedly, with TF-TAVR surpassing SAVR in older patients and approaching similar numbers in low-risk patients.^7^ Yet, long-term TAVR durability data in younger patients remain limited while SAVR continues to show excellent outcomes.^12^ Many centres such as ours have adopted a minimally invasive approach for isolated SAVR, where rapid-deployment valves offer reduced operative times and a favourable recovery. In a Sutureless and Rapid-Deployment Aortic Valve Replacement International Registry (SURD-IR) analysis of 2257 cases, SAVR via thoracotomy was associated with shorter ICU and hospital stays and lower stroke rates.^13^ The CADENCE-MIS trial further showed reduced cross-clamp times in MI-RD-SAVR versus conventional SAVR despite limited access.^14^

Before widespread TAVR adoption, RD MI-SAVR was an attractive option for older low-risk patients, prompting comparisons with TF-TAVR. A GARY registry sub-study investigated early outcomes following TF-TAVR and RD-SAVR in PSM cohorts and showed higher rates of disabling stroke, blood transfusions, and new onset of atrial fibrillation in the RD-SAVR group.^15^ The need for PPI and vascular complications was higher following TF-TAVR and no difference in-hospital mortality was observed. Another group compared TAVR and RD-SAVR in PSM low-risk cohorts and showed no difference in 30 mortality.^16^ However, at 5 years, survival and freedom from major adverse events were lower after TAVR, whereas PPI and PVL ≥ 2 were higher. Our low-risk cohort showed improved early and intermediate-term survival after SAVR. However, the observed inconsistency with our cohort might be attributable to a higher prevalence of full sternotomy (37%) and more patients treated with another platform of sutureless SAVR (41% Edwards Intuity vs 61% Perceval valve), reflecting a less selected patient population and centres with limited experience in minimally invasive approaches. As expected, the SAVR cohort had longer procedural times and hospital stays compared with TF-TAVR.

A recent PSM including 16 studies compared 3258 patients receiving sutureless SAVR with 3258 patients receiving TAVR. Similar to our study, a significantly higher mortality in the TAVR group at 1 year [*RR *= 0.53, 95% CI (0.32, 0.87) and 5 years [*RR *= 0.56, 95% CI (0.46, 0.70), *I*^2^ = 0%, *P *< .01] was observed. Moreover, the SAVR cohort had lower rates of moderate-severe PVL (*P* < .01) and PPI (*P* = .04), but higher rates of major bleeding events (*P* < .01) and new onset of atrial fibrillation (*P* < .01).^17^

Improved survival in favour of SAVR, which has never been observed in the well-known RCTs, is not confined to the RD-valves versus TAVR comparison alone. In their meta-analysis (3 RCTs and 5 PSM studies conducted until 2022), including 5444 patients, Sá et al^8^ showed a higher risk of all-cause mortality after TAVR compared with SAVR (all types) at 8-year follow-up. Survival after TAVR was non-inferior in the first 2 years, however, decreased beyond this timeframe. Interestingly, the difference was driven by the PSM studies and not by the RCTs. A recent STS database analysis revealed increased TAVR rates in patients ≤60 years from 7.2%-45.7% from 2013 through 2021, with an annual increase of 4.7%.^9^ In this collective, TAVR was associated with increased mortality at 5-year follow-up (HR 2.5, *P* = .02). These findings are not confined to the U.S.; data from Europe paint a similar picture. The Austrian registry AUTHEARTVISIT included 18 882 patients ≥65 years old treated with TAVR or biological SAVR and observed a significantly reduced overall survival in the TAVR group in both un-matched and PSM cohorts beyond 1-year follow-up.^18^

The discrepancy between RCTs and PSM studies comparing SAVR vs TAVR might indicate a more fundamental problem. Patients enrolled in RCTs present highly selected cohorts, as for instance, more than 30 exclusion criteria were present in the Partner 3 trial.^5^ Alperi et al^19^ showed that almost half of real-world low-risk AS patients express at least one exclusion criterion from one of the important TAVR vs SAVR RCTs.

Also, a significant proportion of patients included in major RCTs comparing SAVR vs TAVR was subject to deviation from assigned randomized treatment, loss of follow-up, and receipt of additional procedures.^20^ There was an imbalance of these factors, which were all more present in the SAVR trial arms. These findings may threaten the internal validity of the investigated studies, as they have a risk of introducing attrition and performance bias. Despite the aforementioned risks of systematic bias within RCTs, it is important to remember that retrospective PSM studies cannot achieve the same level of matching of treatment as randomization does, as key parameters like frailty indices might not have been measured preoperatively in all patients.

The patho-mechanism behind increased mortality in TAVR cohorts at intermediate to long-term follow-up could be linked to a higher incidence of NSVD, PPI, and thromboembolic events after TAVR, also observed in our cohort.^21^^,^^22^ Additionally, SVD after TAVR is becoming more relevant as TAVR is moving into the younger patients. Data from registries such as the EXPLANT-TAVR cohort indicated a high operative mortality after TAVR explantation, with a large proportion of individuals requiring complete root replacement, possibly affecting long-term survival.^23^

### Limitations

This observational study lacked a centre-independent assessment of adverse events and an independent core lab for echocardiographic follow-up. Although PSM yielded comparable and well-matched cohorts, a slight EuroScore imbalance remained. This is not a prospective randomized trial, and clinical selection bias due to other factors might be present, especially frailty indices. Within this analysis, we compared 2 types of procedures (RD MI-SAVR vs TF-TAVR); within the surgical study cohort, only a single valve model was used, whereas in the transcatheter cohort, several valve models were present.

## CONCLUSIONS

Minimally invasive AVR with RD valves represents an excellent treatment modality for severe AS in an older, low-risk patient cohort and was associated with a higher survival and reduced number of adverse events in comparison to TF-TAVR. The results of this PSM cohort are in line with several other real-world cohorts and diverge substantially from evidence known from larger RCTs. Randomized trials comparing minimally invasive SAVR vs TAVR with longer follow-up and less-selective exclusion criteria are needed to further investigate survival differences in low-risk patients with AS.

## Supplementary Material

ivag007_Supplementary_Data

## Data Availability

The data underlying this article will be shared on reasonable request to the corresponding author.
