# Supplementary material for: Transcatheter Versus Minimally Invasive Surgical Aortic Valve Replacement With Rapid-Deployment Valves: A Propensity-Matched Analysis
Source: Interdiscip Cardiovasc Thorac Surg. 2026 Feb 17;41(2):ivag007. doi: 10.1093/icvts/ivag007 (PMC12953240; doi:10.1093/icvts/ivag007)
Supplement: ivag007_Supplementary_Data [file ivag007_supplementary_data.pdf]

Suppl. Table 1: A comparison between TAVR und SAVR groups in the matched and in the unmachted population are given by means and Standardized mean differences and corresponding loveplot.

Variable	Treated (Unmatched) mean	Control (Unmatched) mean	SMD (Unmatched)	Treated (Matched) mean	Control (Matched) mean	SMD (Matched)
Age	72.575	79.986	-0.998	76.033	76.945	-0.123
Sex	0.497	0.480	0.036	0.497	0.519	-0.044
BMI	28.233	27.359	0.165	27.617	27.782	-0.031
Activeorformersmoker	0.175	0.255	-0.211	0.197	0.202	-0.014
Hypertension	0.848	0.906	-0.163	0.863	0.885	-0.061
Dyslipidemia	0.588	0.816	-0.465	0.716	0.721	-0.011
Diabetes	0.247	0.302	-0.126	0.268	0.268	0.000
PriorMI	0.042	0.080	-0.184	0.049	0.055	-0.027
PriorPCI	0.082	0.261	-0.650	0.120	0.131	-0.040
PriorStroke	0.155	0.104	0.141	0.126	0.126	0.000
Peripheralvasculardisease	0.045	0.108	-0.305	0.049	0.060	-0.053
Atrialfibrillationorflutter	0.133	0.359	-0.669	0.219	0.235	-0.048
Previouspacemakerimplant	0.045	0.133	-0.423	0.082	0.077	0.026
Chronicallungdisease	0.158	0.186	-0.077	0.158	0.164	-0.015
Dialysis	0.007	0.029	-0.244	0.011	0.016	-0.063
Creatinine	0.981	1.330	-0.910	1.035	1.081	-0.119
PorcellainAorta	0.000	0.092	-0.429	0.000	0.000	0.000
EuroSCOREII	1.955	5.531	-2.210	2.609	2.777	-0.104

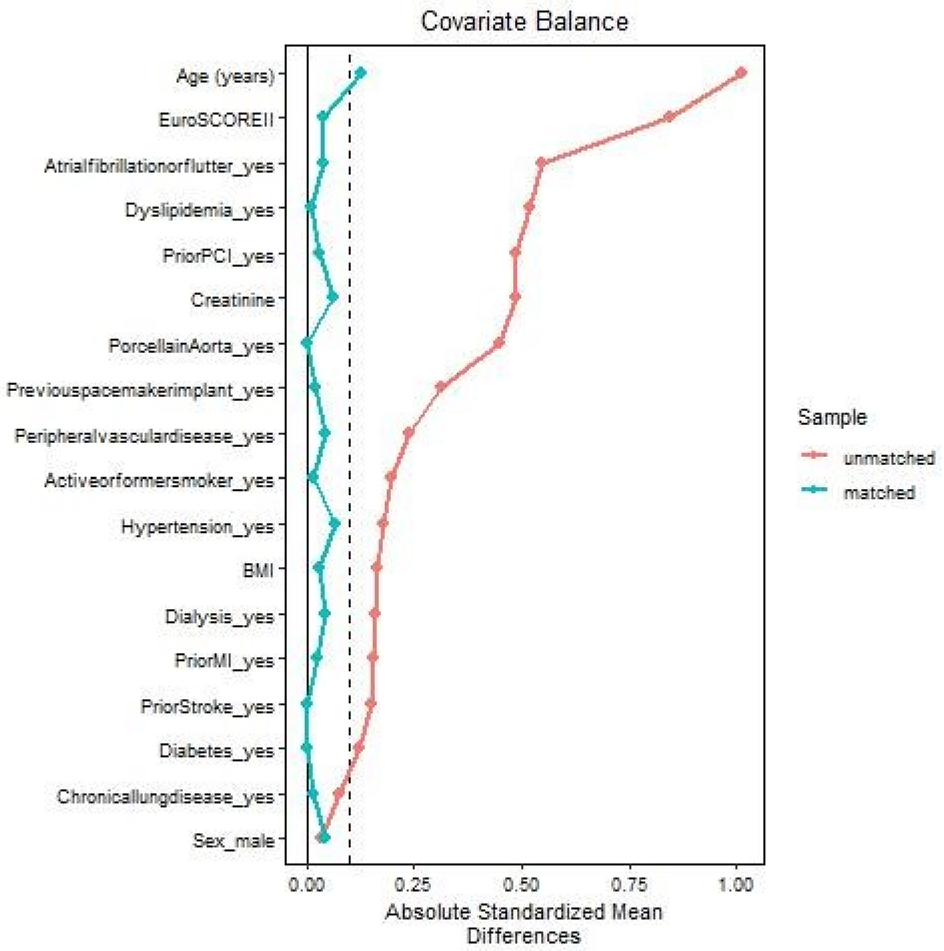

<u>**Suppl Fig. 1:**</u> We assessed overlap by comparing the distributions of the estimated propensity scores (PS) in SAVR vs TAVR before and after matching. The common support was defined as the intersection of the group-specific PS ranges (min–max); as a robustness check we also report a trimmed common support using the 1st–99th percentiles.

Before matching, the common support (min–max) spanned 0.009–0.995. 18.4% of TAVR and 0.2% of SAVR observations lay outside this range (trimmed common support 0.059–0.921). After 1:1 nearest-neighbour matching, the common support (min–max) remained 0.009–0.995, and only 0.5% of TAVR and 0.5% of SAVR observations were outside the common support, indicating adequate overlap post-matching.

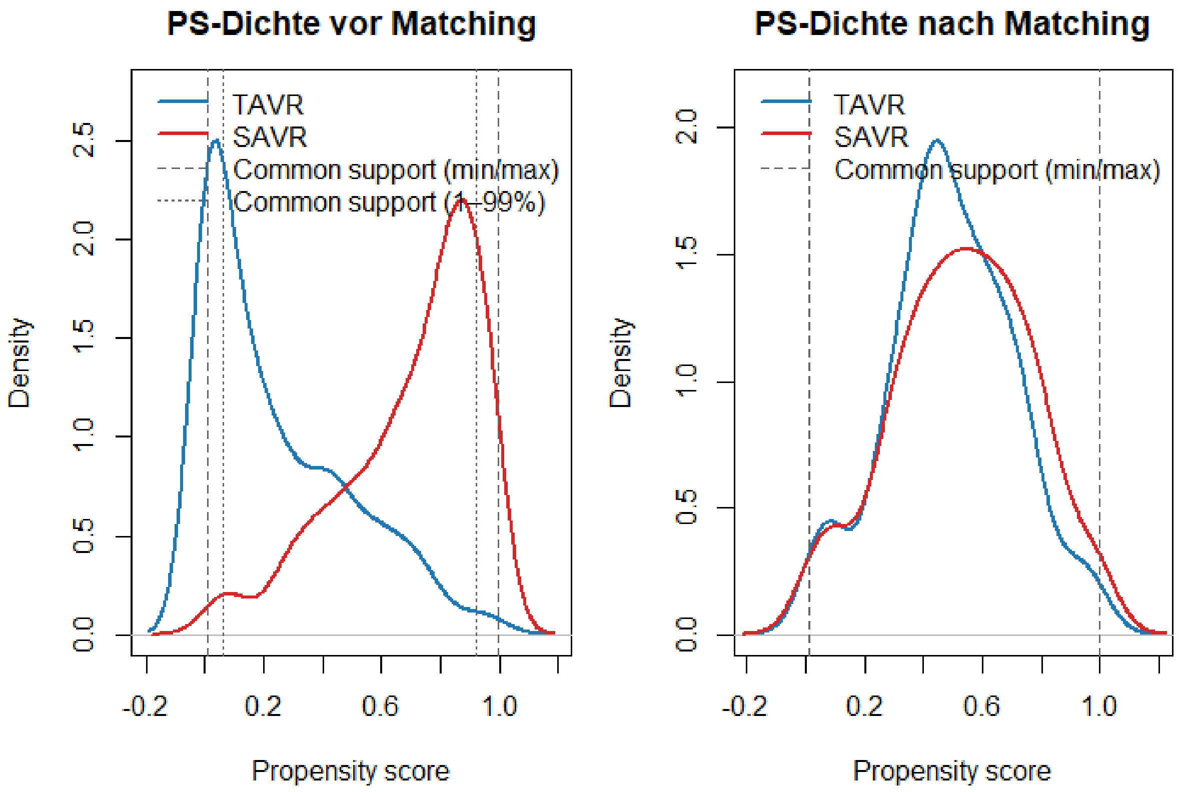

<u>Suppl. Fig. 2:</u> Propensity-score distributions for SAVR and TAVR before (left) and after (right) matching are shown. Dashed lines indicate the min–max common support; dotted lines show the 1st–99th percentile trimmed support (pre-match only).

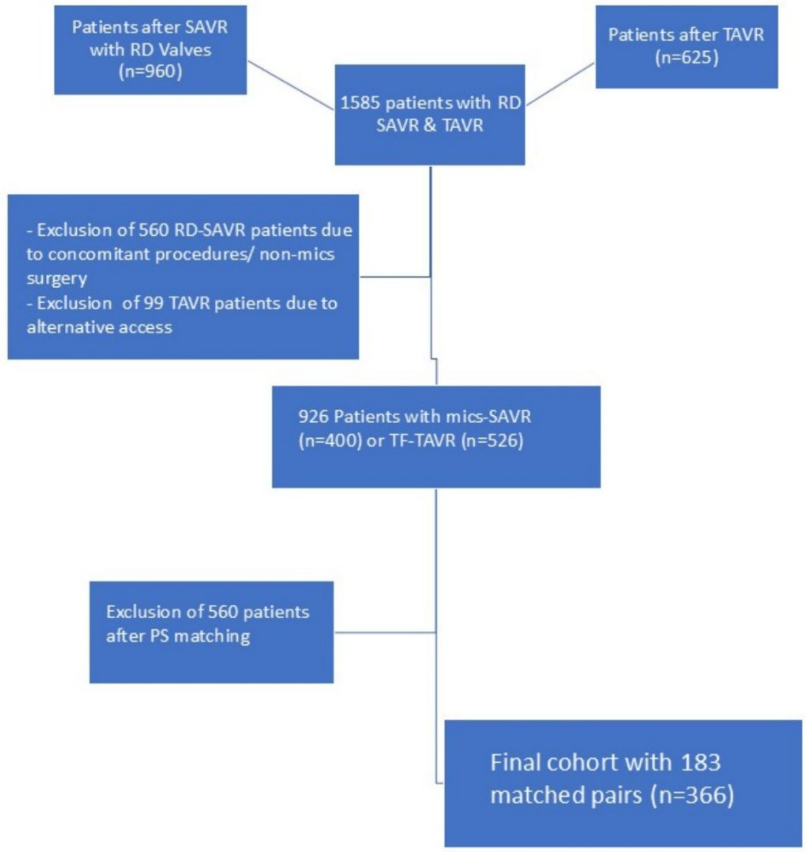

<u>Suppl. Fig. 3:</u> Patient flow chart illustrating how the study cohort was obtained
